# Efficient Synthesis of (*R*)-(+)-Perillyl Alcohol From (*R*)-(+)-Limonene Using Engineered *Escherichia coli* Whole Cell Biocatalyst

**DOI:** 10.3389/fbioe.2022.900800

**Published:** 2022-04-25

**Authors:** Chao Sun, Rubing Zhang, Congxia Xie

**Affiliations:** ^1^ A State Key Laboratory Base of Eco-Chemical Engineering, College of Chemistry and Molecular Engineering, Qingdao University of Science and Technology, Qingdao, China; ^2^ CAS Key Laboratory of Bio-Based Materials, Qingdao Institute of Bioenergy and Bioprocess Technology, Chinese Academy of Sciences, Qingdao, China

**Keywords:** whole cell catalysis, (*R*)-(+)-perillyl alcohol, NADH regeneration, alkL, *escherichia coli*

## Abstract

(*R*)-(+)-perillyl alcohol is a much valued supplemental compound with a wide range of agricultural and pharmacological characteristics. The aim of this study was to improve (*R*)-(+)-perillyl alcohol production using a whole-cell catalytic formula. In this study, we employed plasmids with varying copy numbers to identify an appropriate strain, strain 03. We demonstrated that low levels of alKL provided maximal biocatalyst stability. Upon determination of the optimal conditions, the (*R*)-(+)-perillyl alcohol yield reached 130 mg/L. For cofactor regeneration, we constructed strain 10, expressing FDH from *Candida boidinii*, and achieved (*R*)-(+)-perillyl alcohol production of 230 mg/L. As a result, 1.23 g/L (*R*)-(+)-perillyl alcohol was transformed in a 5 L fermenter. Our proposed method facilitates an alternative approach to the economical biosynthesis of (*R*)-(+)-perillyl alcohol.

## Introduction

(*R*)-(+)-limonene is a ubiquitous natural monocyclic monoterpene that accounts for over 90% of orange peel oil (Bicas et al., 2009). This makes it a readily available and affordable ingredient for fine chemical production (Berger et al., 1999). For the past few years, people considered using (*R*)-(+)-limonene to produce biological flavors. (*R*)-(+)-perillyl alcohol, one of its oxygenated derivatives, is a stable alcohol that is often used in anti-cancer drugs, such as colon, skin, head and neck (Gupta et al., 2005; Chen et al., 2006; Peffley et al., 2007; Yeruva et al., 2007; Das et al., 2010; Sobral et al., 2014).

In recent years, (*R*)-(+)-perillyl alcohol synthesis, particularly using microbial fermentation (MF) and enzymatic conversion (EC), has garnered much attention. The MF approach utilizes genetic engineering of the mevalonate pathway to simultaneously promote cell proliferation and (*R*)-(+)-perillyl alcohol synthesis. Until recently, it was reported that the content of glucose-derived (*R*)-(+)-perillyl alcohol, by artificially engineered *Escherichia coli*, was extremely low (87 mg/L) (Sun et al., 2021). This may be due to the complicated metabolic pathways, byproduct toxicity, and metabolic burden (Marmulla and Harder, 2014; Zhu et al., 2021). Thus, it is necessary to develop a new, simple, and efficient method for (*R*)-(+)-perillyl alcohol production.

EC methods include cell-free enzymes and whole cells (Li et al., 2020). Employing whole cells eliminate the need for cell-free enzyme purification and separation. Moreover, whole cells are more stable and resistant to environmental stimuli than free enzymes (Song et al., 2015; Yang et al., 2019). Despite limited investigations on the whole-cell bioconversion of (*R*)-(+)-limonene to (*R*)-(+)-perillyl alcohol, this approach offers much promise owing to the successful and efficient conversion of other limonene-derived monoterpenes using whole cell catalysts. Fortunately, the limonene-derived biotransformation of other monoterpenes have been reported in *Penicillium* sp. (Rottava et al., 2010; Badee et al., 2011; Molina et al., 2013), *Aspergillus* sp. (García-Carnelli et al., 2014), *Fusarium oxysporum* (Bicas et al., 2010a), *Yarrowia lipolytica* ATCC 18942 (Ferrara et al., 2013), *Colletotrichum nymphaeae* (Sales et al., 2018), *Pseudomonas fluorescens* (Chatterjee and Bhattacharyya, 2001; Bicas et al., 2008; Mirata et al., 2009; Soares-Castro et al., 2017) and *Sphingobium* sp. (Bicas et al., 2010b). (*R*)-(+)-α-terpineol synthesis from (*R*)-(+)-limonene using *Sphingobium* sp., with a Plackett-Burman design, followed by a Central Composite Design, gave a yield of 240 g/L after 96 h (Molina et al., 2019), which was the highest reported monoterpene yield. Mirata et al. (2009) also demonstrated that *Pseudomonas putida* DSM 12264 oxidizes the C7 site of limonene to produce (*R*)-(+)-perillic acid as the main product. Moreover, the perillic acid content was improved using *in situ* separation technology, thus, producing a yield of 31 g/L after 7 days. In addition, whole-cell reactions and subsequent purifications are relatively easy to perform, relative to the direct fermentation process (Oh et al., 2015).

**FIGURE 1:**
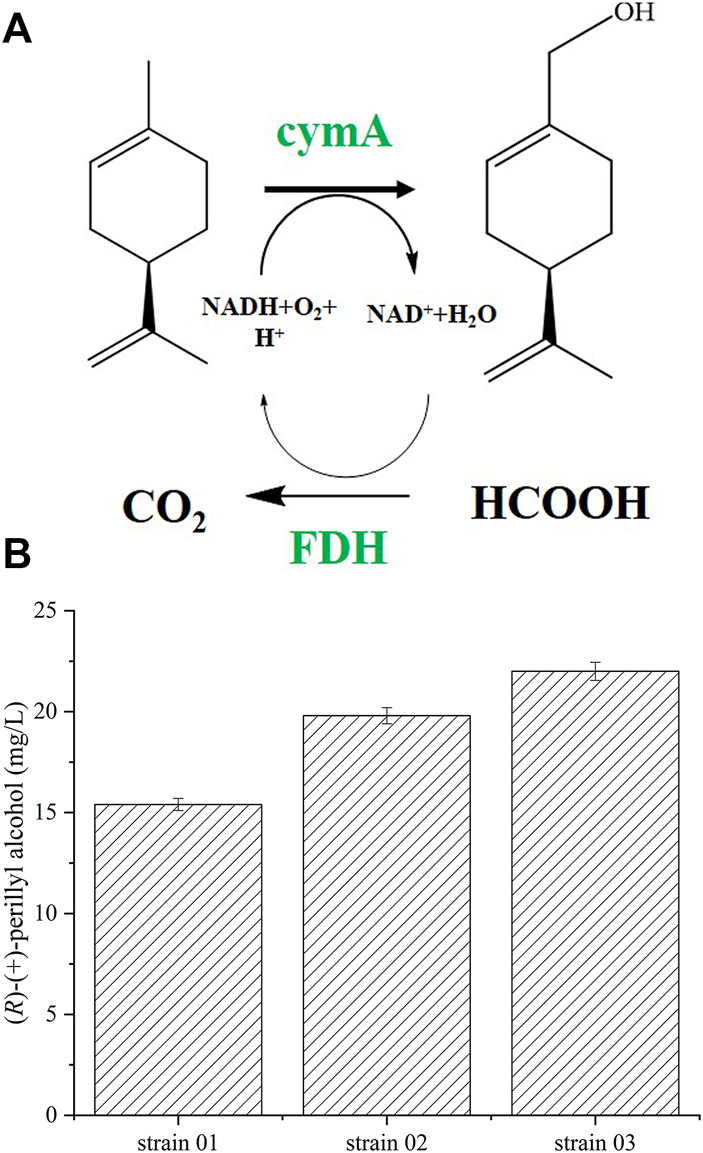
(*R*)-(+)-perillyl alcohol synthesis using whole cell biocatalysts. **(A)** (*R*)-(+)-perillyl alcohol biosynthesis from (*R*)-(+)-limonene, using a whole cell system. **(B)** (*R*)-(+)-perillyl alcohol production with different plasmids. Reaction conditions (20 ml): whole cell catalysts (OD_600_ = 30), 25 μl (*R*)-(+)- limonene, 8.0 ml dioctyl phthalate (DINP), and PBS buffer (50 mM, pH 7.4) at 30°C for 6 h. Data expressed as mean ± s.d. (*n* = 3). Abbreviations: CymAa, p-cymene monoxygenase hydroxylase, CymAb, p-cymene monoxygenase reductase, CymA, CymAa and CymAb, FDH, formate dehydrogenase.

In this study, we co-expressed p-cymene monooxygenase hydroxylase (cymAa) and p-cymene monoxygenase reductase (cymAb) in *E. coli*. Doing so, we converted (*R*)-(+)-limonene to (*R*)-(+)-perillyl alcohol (Figure 1A). Firstly, the catalytic activity was improved by expressing plasmids of varying copies. Moreover, alkL overexpression enhanced hydrophobic substrate uptake, whereas, low alkL level augmented whole cell stability. Next, we optimized biotransformation kinetics by altering parameters like initial pH, temperature, whole cell biocatalyst, and substrate concentration, to achieve the highest (*R*)-(+)-perillyl alcohol concentration. Employing the optimal conditions, along with formate dehydrogenase (FDH) for NADH regeneration, the strain 10 produced 1.23 g/L (*R*)-(+)-perillyl alcohol for 20 h in a 5 L fermenter. Our novel approach to (*R*)-(+)-perillyl alcohol synthesis was demonstrated to be highly efficient and green, with great potential for large-scale synthesis of monoterpenoids.

## Materials and Methods

### Strains and Culture Conditions

Table 1 lists the strains employed in our research. Bacterial cultures were grown in LB medium (per liter: 10 g tryptone, 5 g yeast extract and 10 g NaCl) with 10 g/L glucose during shake-flask fermentation. The bioreactor fermentation medium contained (per liter) 8.7 g glycerol, 10 g tryptone, 11 g yeast extract, 2.4 g (NH_4_)SO_4_, 2 g citric acid. H_2_O, 20.6 g Na_2_HPO_4_.12H_2_O, 5.22 g KH_2_PO_4_, and 0.12 g MgSO_4_. A feed solution (50% glycerol, 5 g/L yeast extract and 5 g/L tryptone) was utilized. We also introduced supplemental antibiotics (100 μg/ml ampicillin, or 50 μg/ml kanamycin) to maintain corresponding plasmids.

**TABLE 1:** Strains and plasmids used in this study.

Name	Relevant Characteristics	References
Strains		
*E.coli* DH5α	F^−^ *rec*A *endA*1*Φ80dlac*Z*△M15hs*dR17(r_k_^-^m_k_^+^)λ^−^	Invitrogen
*E.coli* BL21 (DE3)	F- ompT hsdSB (rB – mB - ) gal dcm rne131 λ(DE3)	Invitrogen
*E.coli* JM109 (DE3)	endal glnv44 thi^−1^ relal gyra96 recal mcrB^+^△(lac-proAB) e14^−^[F, traD36 proAB^+^ lacZ △M15] hsdr17(rk^−^ mk^+^) λ(DE3)	Invitrogen
*E.coli* Rosstta (DE3)	F-ompT hsdSB(rB-mB-)gal dcm(DE3)	Invitrogen
*E.coli* MG1655 (DE3)	F- λ- ilvg- rfb-50 rph-1λ(DE3)	Invitrogen
srain 01	*E.coli.* BL21(DE3)/pACYCDuet-2-*cymA*	This study
srain 02	*E.coli.* BL21(DE3)/pET28a(+)-*cymA*	This study
srain 03	*E.coli.* BL21(DE3)/pRSFDuet-1-*cymA*	This study
srain 04	*E.coli.* BL21(DE3)/(pRSFDuet-1-*cymA and* pACYCDuet-2-lac*-alkL*)	This study
srain 05	*E.coli.* BL21(DE3)/(pRSFDuet-1-*cymA and* pACYCDuet-2-trc*-alkL*)	This study
srain 06	*E.coli.* BL21(DE3)/(pRSFDuet-1-*cymA and* pACYCDuet-2*-alkL*)	This study
srain 07	*E.coli.* JM109 (DE3)/(pRSFDuet-1-*cymA and* pACYCDuet-2-lac*-alkL*)	This study
srain 08	*E.coli.* Rosstta (DE3)/(pRSFDuet-1-*cymA and* pACYCDuet-2-lac*-alkL*)	This study
srain 09	*E.coli.* MG1655 (DE3)/(pRSFDuet-1-*cymA and* pACYCDuet-2-lac*-alkL*)	This study
srain 10	*E.coli.* MG1655 (DE3)/(pRSFDuet-1-*cymA-fdh and* pACYCDuet-2-lac*-alkL*)	This study
Plasmids		
pRSFDuet-1	double T7 promoters, RSF ori, KanR	Novagen
pET28a(+)	Kan^r^oripBR322lacI^q^T7p	Novagen
pACYCDuet-1	Cm^r^ p15A lacI T7lac	Novagen
pACYCDet-2	Amp^r^ p15A lacI T7lac	This study
pACYCDet-2-*cymA*	pACYCDet-2 carrying *cymAa* and *cymAb* from *Pseudomonas putida*	This study
pET28a(+)-*cymA*	pET28a(+) carrying *cymAa* and *cymAb* from *Pseudomonas putida*	This study
pRSFDuet-1-*cymA*	pRSFDuet-1 carrying *cymAa* and *cymAb* from *Pseudomonas putida*	This study
pACYCDet-2-lac-*alkL*	pACYCDuet-2 carrying *alkL* from *P. putida GPo1,* the *alkL* gene with lac promoter	This study
pACYCDet-2-trc-*alkL*	pACYCDuet-2 carrying *alkL* from *P. putida GPo1,* the *alkL* gene with trc promoter	This study
pACYCDet-2-*alkL*	pACYCDuet-2 carrying *alkL* from *P. putida GPo1,* the *alkL* gene withT7 promoter	This study
pRSFDuet-1-*cymA-fdh*	pRSFDuet-1 carrying *cymAa* and *cymAb* from *Pseudomonas putida, fdh* from *Candida boidinii*	This study

### Plasmid Construction

First, we codon optimized *cymAa* and *cymAb* from *Pseudomonas putida* (Sun et al., 2021). Next, they were cloned into pACYCDuet-2 using *Bam*HI/*Sac*I restriction sites to construct pACYCDuet-2-*cymA*. The pET28a(+)-*cymA* and pRSFDuet-1-*cymA* plasmids were constructed in a similar fashion. The *alkL* gene (GenBank: AJ245436.1) from *Pseudomonas putida* GPo1 and *fdh* gene (GenBank: AJ011046.2) were codon optimized by BGI and produced by GeneWiz (Suzhou, China), before cloning into PUC57. The *alkL* gene was assembled into the pACYCDuet-2 plasmid using C115 (Vazyme Biotech Co., Ltd., Nanjing, China) at the *Nco*I/*Sac*I sites, thus, generating the pACYCDuet-2-*alkL* plasmid. The *fdh* gene from *Candida boidinii* was then ligated into pRSFDuet-1-*cymA* to create pRSFDuet-1-*cymA-fdh.* All primers are shown in Supplementary Table S1.

### Expression of Recombinant Proteins in *E. coli* Strains

First, monoclonal strain was selected from the LB agar plate and cultured in a tube with 4 ml LB broth till the exponential phase. Next, seed cultures were inoculated into 500 ml baffled triangular flask carrying 100 ml LB and 10 g/L glucose at 37°C, 200 pm with an OD_600_ of 0.6–0.8, the temperature was set to 16°C, and induction was done with 0.2 mM IPTG for 20 h.

The recombinant *E. coli* were collected by centrifugation (8000 g for 10 min), PBS-rinsed two times (50 mM PBS buffer, pH 7.4), and resuspended to obtain a whole-cell catalyst, followed by storage at 4°C until further analysis.

### Reaction Condition Optimization

All optimization experiments were performed in 250 ml flasks with 20 ml reaction broth, whole-cell catalysts (OD_600_ = 30), 25 μl (*R*)-(+)- limonene, 8.0 ml dioctyl phthalate (DINP), and 50 mM PBS buffer (pH 7.4). All reactions were carried out for 6 h at 30°C, with stirring at 200 rpm.

The variables examined included pH, reaction temperature, NADH, cell concentrations, and substrate concentrations. To optimize pH, experiments were conducted in several buffers (pH 5.8–8.0). Temperature optimization was done by monitoring whole-cell conversion activity between 16 and 37°C. To optimize NADH concentrations, 0, 1, and 2 mM NADH were examined. To establish optimal cell concentration, whole cell catalysts between OD_600_ 10–70 were used. Moreover, following optimization of the above conditions, the effects of different substrate concentrations (1–40 g/L) were examined. Lastly, different ammonium formate concentrations (10–100 g/L) were assessed for optimal reaction results.

### Conversion of (*R*)-(+)-Limonene Into (*R*)-(+)-Perillyl Alcohol Using Whole Cell Biocatalyst in a Bioreactor

The engineered strain was cultured for 12 h at 37°C in 250 ml shake flask with 50 ml LB medium. Fed-batch fermentations were applicated in a 5 Lfermenter (ez-control, Applikon) containing 2 L fermentation broth at 0.5–2 vvm aeration and 400–800 rpm, respectively. Subsequently, the fermentation was inoculated with 5.0% (v/v) seed culture, and performed at 37°C and pH 7.0 with the addition of NH_4_OH (25%, v/v). When the OD_600_ reached 15 (initial glycerol was consumed), 0.2 mM IPTG was introduced and the temperature was set to 16°C for 20 h. The feeding speed was variable for the induction period with 50% glycerol supplement (1.5–2.0 g/L.h). Dissolved oxygen (DO) levels were not less than 20%. The recombinant *E. coli* were then collected *via* centrifugation (8000 g for 10 min), PBS-rinsed two times (50 mM, pH 7.4), and resuspended to obtain the whole-cell catalyst, followed by storage at 4°C.

Under optimized reaction conditions, we employed whole-cell biocatalyst to synthesize (*R*)-(+)-perillyl alcohol in a 5 L fermenter. The conversion mixture (1 L) included 20 g/L (*R*)-(+)-limonene, 40 g/L ammonium formate, recombinant cells (OD_600_ = 50), 40 ml dioctyl phthalate (DINP), and PBS buffer (50 mM, pH 7.4), with constant stirring at 20°C for 24 h. (*R*)-(+)-limonene and (*R*)-(+)-perillyl alcohol were detected every 4 h.

### The Whole Cell Catalyst Activity Assessment

The whole-cell catalyst enzymatic activity was determined by adding 2 mM (*R*)-(+)-limonene and whole-cell catalyst (0.5 g_CDW_.L^−1^), followed by detection of (*R*)-(+)-perillyl alcohol concentration. The reaction (1 ml) was carried out in triplicate at 30°C for 10 min and 200 rpm (Cornelissen et al., 2011). Activities are presented in 1 Ug_CDW_^−1^, with 1 Ug_CDW_^−1^ = 1 μmol product min^−1^ g_CDW_^−1^.

### Analytical Conditions

The *E. coli* biomass was measured using OD_600_ on a spectrophotometer (Cary 50 UV-vis, Varian). Cell Dry weight (CDW) was calculated as follows: 1 OD_600_ = 0.323 g_CDW_.L^−1^. The Shimadzu GC-MS system (TQ8050) was employed to detect (*R*)-(+)-limonene, (*R*)-(+)-perillyl alcohol, and (*R*)-(+)-perillyl aldehyde, using the following conditions: 30 m DB-5MS column. (inside diameter 0.32 mm, film thickness 0.25 μm); temperature: held at 50°C, before ramp up 10°C/min to 250°C, followed by another hold at 250°C for 10 min. Highly pure helium at a linear velocity of 1 ml/min served as the carrier; the injector temperature was set to 250°C; a split ratio was adjusted to 1:10; ion source temperature was set to 230°C, and mass range was set to m/z 40–500. The fermentation broth was mixed with equal volume of ethyl acetate before spinning. The organic layer was further assessed using specific test methods and sample treatments, as reported earlier (Sun et al., 2021).

## Results

### Recombinant (*R*)-(+)-Perillyl Alcohol Synthesis Using Different Expression Systems

In a previous report, (*R*)-(+)-perillyl alcohol was obtained in an engineered *E. coli* by expressing *cymA* genes from glucose (Sun et al., 2021). Using this technique, strains 01, 02, and 03 were constructed (Table 1). In prior research, enzyme expression was balanced using plasmids with varying copy numbers (Hou et al., 2017). In this study, we employed three plasmids with distinct copy numbers: pRSFDuet-1 (100 copies per cell), pET28a (+) (40 copies per cell), and pACYCDuet-1 (10 copies per cell) (Rico et al., 2019). Next, we assessed their synthetic ability and enzymatic activity (Figure 1B and Supplementary Table S2). Relative to strain 03, the specific activities of cymA in strains 01 and 02 were 2.56 and 2.89 U g_CDW_^−1^, respectively, and they decreased by 32 and 24%. Based on our analysis, strain 03, constructed by pRSFDuet-1 (high copy number), provided the largest yield of (*R*)-(+)-perillyl alcohol at 22.0 mg/L (all units presented as the total fermentation volume, mg/L_tot_).

### Effect of alkL Protein Levels on (*R*)-(+)-Perillyl Alcohol Production

**FIGURE 2:**
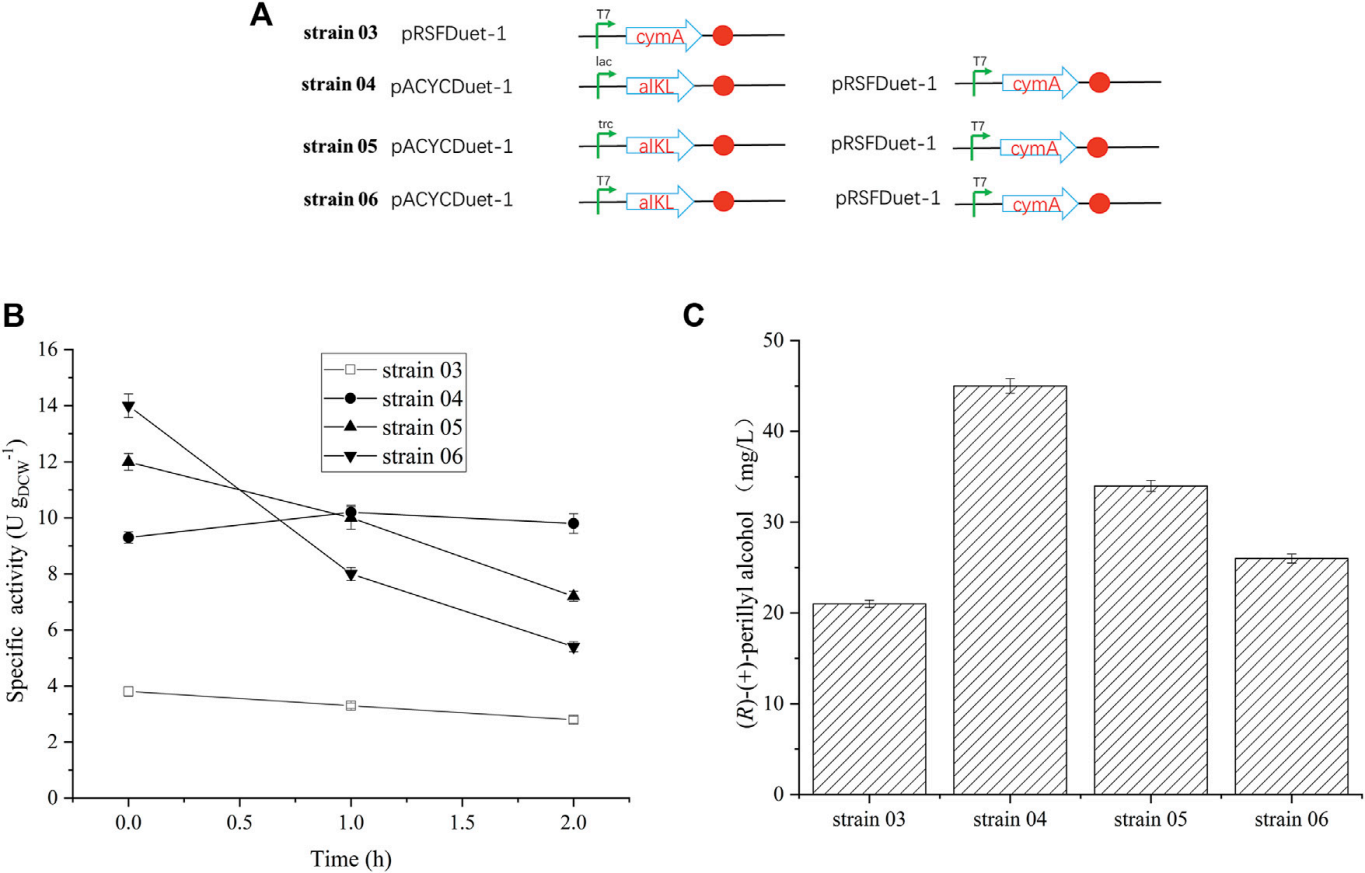
Effect of alkL protein expression on (*R*)-(+)-perillyl alcohol production. **(A)** Strains constructed by different promotors. **(B)** Whole-cell activity assays related to alkL expression. **(C)** Comparison of (*R*)-(+)-perillyl alcohol production in each strain. Reaction conditions (20 ml): whole cell catalysts (OD_600_ = 30), 25 μl (*R*)-(+)- limonene, 8.0 ml dioctyl phthalate (DINP), and PBS buffer (50 mM, pH 7.4) at 30°C for 6 h. Data expressed as mean ± s.d. (*n* = 3).

Earlier reports suggested that alkL enhances uptake and oxygenation of substrate, such as, alkanes, FAMEs, located in the inner membrane or in the cytosol (Julsing et al., 2012; Cornelissen et al., 2013; Grant et al., 2014). To elucidate the outcome of varying alkL levels on (*R*)-(+)-limonene conversion rates and product synthesis kinetics, recombinant *E. coli* BL21 (DE3), with different promoters, were used to construct three distinct strains (strain 04, strain 05, and strain 06) (Table 1 and Figure 2). Predictably, the alkL-negative strain 03 produced the least specific (*R*)-(+)-limonene hydroxylation activity (Figure 2B). In contrast, all alkL-positive strains exhibited markedly higher activities, and the level of activity corresponded to the level of alkL. AlkL-positive strain 06 (medium to high alkL expression) initially produced the maximum specific (*R*)-(+)-limonene hydroxylation activity at 12–14 U g_DCW_^−1^, followed by a rapid decrease. Conversely, strain 04, carrying a low alkL expression, produced relatively stable activity at 10 U g_DCW_^−1^ (Figure 2B), and maintained 45 mg/L (*R*)-(+)-perillyl alcohol, which reached 1.7 times higher than strain 06, with high alkL level (Figure 2C).

### (*R*)-(+)-Perillyl Alcohol Biosynthesis Using Various *E. coli* Strains and Whole Cell Biocatalysts

**FIGURE 3:**
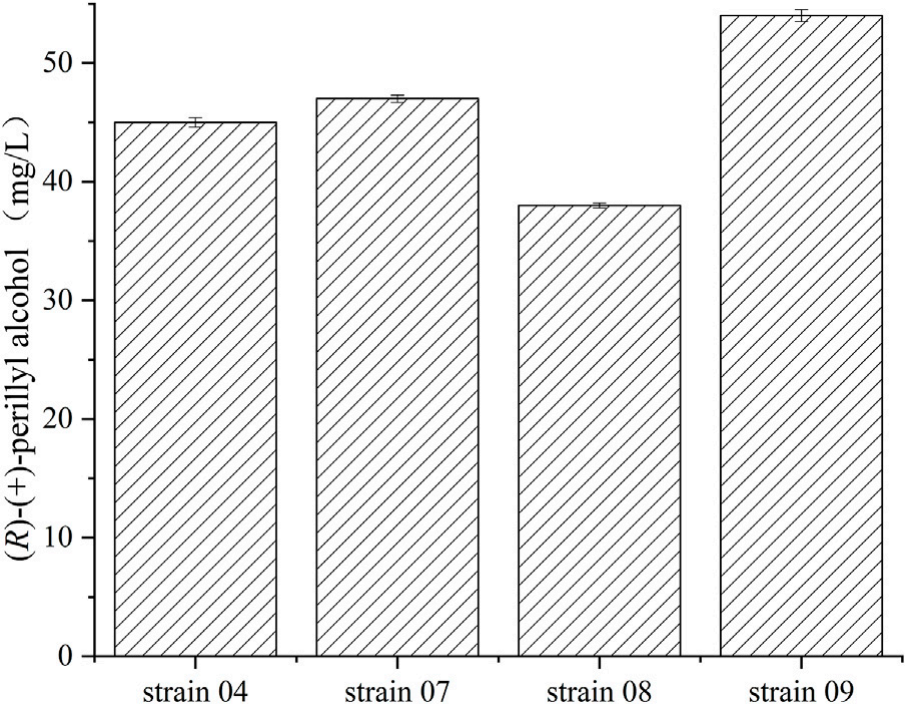
Various concentrations of (*R*)-(+)-perillyl alcohol production by different *E. coli* strains. Reaction conditions (20 ml): whole cell catalysts (OD_600_ = 30), 25 μl (*R*)-(+)- limonene, 8.0 ml dioctyl phthalate (DINP), and PBS buffer (50 mM, pH 7.4) at 30°C for 6 h. Data expressed as mean ± s.d. (*n* = 3).

Recombinant *E.coli.* BL21 (DE3), *E.coli.* JM109 (DE3), *E.coli* Rosstta (DE3), and *E.coli* MG1655 (DE3), harboring both pRSFDuet-1-*cymA* and pACYCDet-2-lac*-alkL*, were examined, in terms of (*R*)-(+)-perillyl alcohol synthesis, in PBS buffer containing 1 g/L of (*R*)-(+)-limonene (Figure 3). The highest concentration was achieved by strain 09, yielding 54 mg/L (*R*)-(+)-perillyl alcohol, which was 1.2 times higher than strain 04. Thus, strain 09 was selected for subsequent investigations on (*R*)-(+)-perillyl alcohol synthesis.

### Optimizing (*R*)-(+)-Perillyl Alcohol Sythesis Reaction Conditions

We further optimized reaction conditions to produce the largest yield of (*R*)-(+)-perillyl alcohol from (*R*)-(+)-limonene. The assessed parameters included initial pH, temperature, NADH introduction, and whole-cell catalysts.

### Optimizing pH and Temperature of Whole Cell Catalysis

**FIGURE 4:**
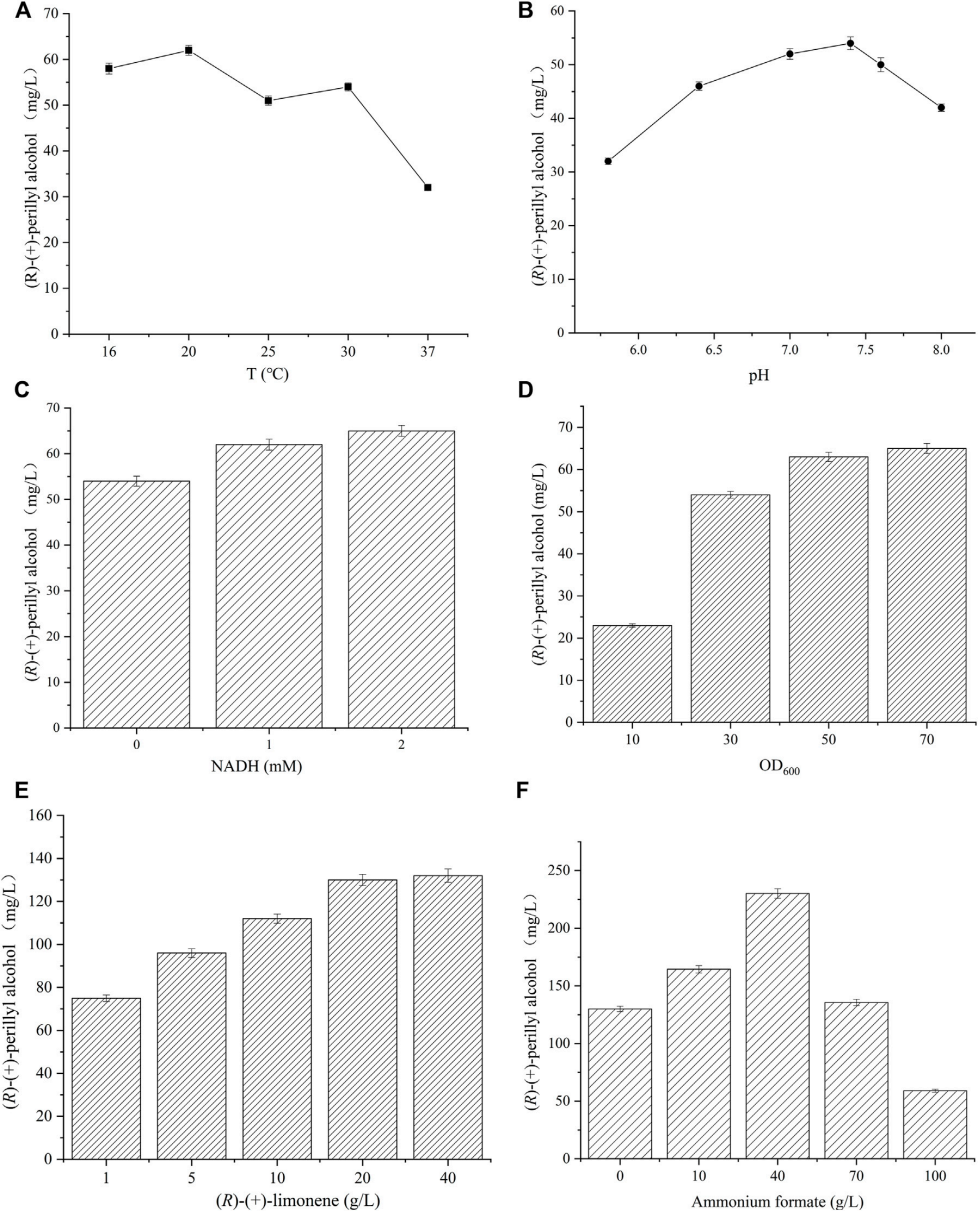
Reaction condition optimization for (*R*)-(+)-perillyl alcohol synthesis. The effect of **(A)** varying pHs and **(B)** temperatures on (*R*)-(+)-perillyl alcohol synthesis. Reaction conditions (20 ml) for different pHs: whole cell catalysts (OD_600_ = 30), 25 μl (*R*)-(+)- limonene, 8.0 ml dioctyl phthalate (DINP), and PBS buffer (50 mM, pH 5.8–8.0) at 30°C for 6 h. Reaction conditions (20 ml) for different temperatures: whole cell catalysts (OD_600_ = 30), 25 μl (*R*)-(+)-limonene, 8.0 ml dioctyl phthalate (DINP), and PBS buffer (50 mM, pH 7.4) at 16–37°C for 6 h. **(C)** Effects of NADH addition on (*R*)-(+)-perillyl alcohol production. Reaction conditions (20 ml): whole cell catalysts (OD_600_ = 30), 25 μl (*R*)-(+)-limonene, 8.0 ml dioctyl phthalate (DINP), NADH (0–2 mM), and PBS buffer (50 mM, pH 7.4) at 30°C for 6 h. **(D)** Effect of biocatalysts concentrations on (*R*)-(+)-perillyl alcohol production. Reaction conditions (20 ml): whole cell catalysts (OD_600_ = 10, 30, 50, 70), 25 μl (*R*)-(+)-limonene, 8.0 ml dioctyl phthalate (DINP), and PBS buffer (50 mM, pH 7.4) at 30°C for 6 h. **(E)** Whole cell biotransformation of (*R*)-(+)-perillyl alcohol at high (*R*)-(+)-limonene concentration. Reaction conditions (20 ml): whole cell catalysts (OD_600_ = 50), (*R*)-(+)-limonene (1, 5, 10, 20, 40 g/L), 8.0 ml dioctyl phthalate (DINP), 2 mM NADH, and PBS buffer (50 mM, pH 7.4) at 30°C for 6 h. **(F)** (*R*)-(+)-perillyl alcohol production with different ammonium formate concentrations. Reaction conditions (20 ml): whole-cell catalysts (OD_600_ = 50), 500 μl (*R*)-(+)-limonene, 8.0 ml dioctyl phthalate (DINP), ammonium formate (0, 10, 40, 70, 100 g/L), and PBS buffer (50 mM, pH 7.4) at 30°C for 6 h. Data expressed as mean ± s.d. (*n* = 3).

To augment (*R*)-(+)-perillyl alcohol synthesis, pH and temperature of whole cell catalysis were optimized in strain 09. We synthesized (*R*)-(+)-perillyl alcohol in different phosphate buffer (pH 5.8–8.0), and pH 7.4 produced the largest yield (Figure 4A). The examined temperatures were 16, 20, 25, 30, and 37°C, and the temperature the produced the most amount of (*R*)-(+)-perillyl alcohol was 20°C (Figure 4B). Taken together, the optimal conditions for (*R*)-(+)-perillyl alcohol synthesis in strain 09 were pH 7.4 and 20°C.

### Enhancing (*R*)-(+)-Perillyl Alcohol Production by Adding NADH

It is possible that the reductase portion of cymA transfers electrons from NADH to the hydroxylase subunit (Figure 1A), using flavin and ferredoxin moieties (Eaton, 1997; Dutta et al., 2010; Dutta et al., 2012). Thus, NADH is a necessary element for the production of (*R*)-(+)-perillyl alcohol from (*R*)-(+)-limonene (Figure 1A). The optimal NADH, according to our results, was 2 mM, which produced 65 mg/L of (*R*)-(+)-perillyl alcohol (Figure 4C).

### (*R*)-(+)-Perillyl Alcohol Synthesis Using Varying Whole Cell Concentrations

An increasing amount of whole cell (OD_600_ of 10, 30, 50, 70) was introduced to identify the optimal whole cell concentration for efficient (*R*)-(+)-perillyl alcohol production. Based on our analysis, cell concentration with an OD_600_ value of 50 markedly enhanced (*R*)-(+)-perillyl alcohol synthesis, whereas, cell concentration of 70 did not alter the titer significantly (Figure 4D). Moreover, the influence of cell densities exceeding OD_600_ value of 50 was unremarkable, likely due to the lack of dissolved oxygen, which is a critical step in (*R*)-(+)-perillyl alcohol synthesis.

### Enhancing (*R*)-(+)-Perillyl Alcohol Production by Increasing Substrate Concentration and Expressing *fdh*

Based on the above findings, we synthesized 75 mg/L (*R*)-(+)-perillyl alcohol in strain 09, under the proper conditions (Figure 4E). Given that excess substrate can vastly improve volumetric productivity, we investigated different substrate concentrations varying from 1 to 40 g/L (Figure 4E). With 20 g/L (*R*)-(+)-limonene, the (*R*)-(+)-perillyl alcohol production was 130 mg/L, which was 1.7 times higher than the production from 1 g/L (*R*)-(+)-limonene.In addition, (*R*)-(+)-perillyl alcohol accumulation was not obvious, when the substrate exceeded 20 g/L.

NADH addition can obviously increase (*R*)-(+)-perillyl alcohol yield (Figure 4C). Since NADH is costly, we integrated a NADH generating pathway, such as, formate dehydrogenase (FDH) for the regeneration of NADH (Liu et al., 2019; Chen et al., 2021). FDH from *Candida boidinii* was expressed and strain 10 was constructed. NADH regeneration was achieved by adding different concentrations of ammonium formate. When 40 g/L ammonium formate was added, the (*R*)-(+)-perillyl alcohol production was 230 mg/L (Figure 4F). Conversely, when ammonium formate exceeded 40 g/L, the product yield decreased rapidly, likely due to enzyme inactivation in presence of excess ammonium formate.

### (*R*)-(+)-Perillyl Alcohol Biosynthesis *via* Whole Cell Catalysis in a Bioreactor

**FIGURE 5:**
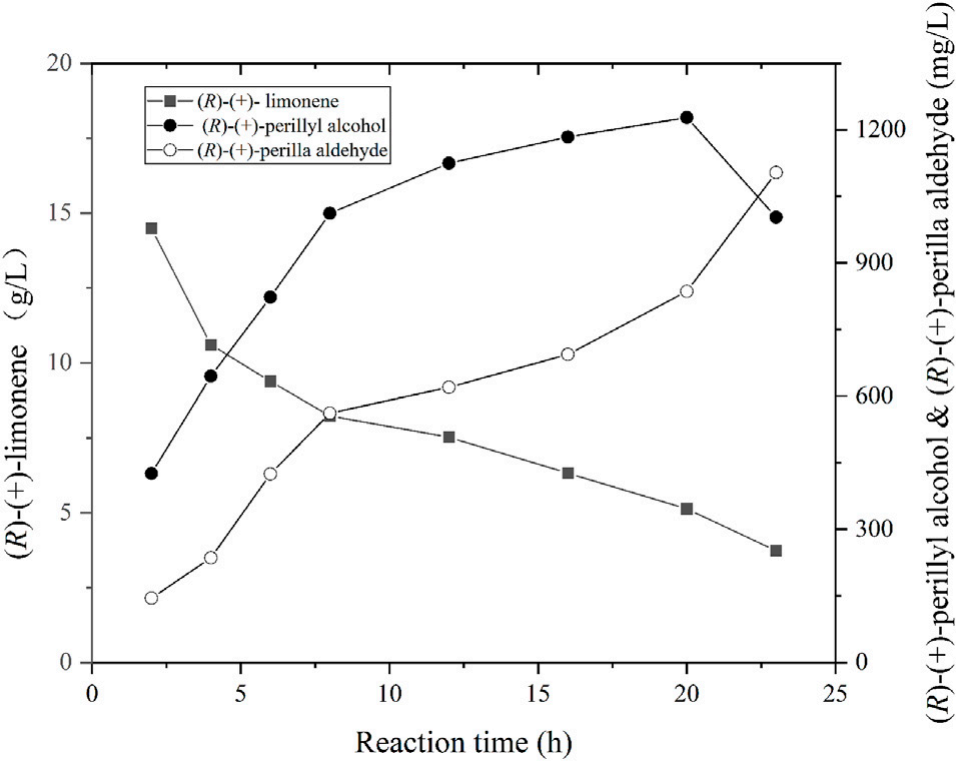
Time course of (*R*)-(+)-limonene hydroxylation using whole cell of strain 10. Reaction conditions (1 L): whole cell catalysts (OD_600_ = 50), 25 ml (*R*)-(+)-limonene, 40 g ammonium formate, 400 ml dioctyl phthalate (DINP), and PBS buffer (50 mM, pH 7.4) at 20°C for 24 h.

Using the optimal parameters from aforementioned experiments (i.e., pH, cell density, and *fdh* gene expression), recombinant *E. coli* strain 10 was cultivated in a 5 L bioreactor. The biomass was obtained *via* centrifugation and employed for the biotransformation of (*R*)-(+)-limonene to (*R*)-(+)-perillyl alcohol. The reaction was conducted as follows: 20°C with 300–800 rpm and 0.5 vvm; pH of 7.4; and recombinant cell concentration OD_600_ of 50. The (*R*)-(+)-perillyl alcohol synthesis yield is shown in Figure 5. In brief, (*R*)-(+)-perillyl alcohol yield rose drastically between 0 and 12 h, then started to decreased between 12 and 20 h, and the titer reduced even more after 20 h. Our largest (*R*)-(+)-perillyl alcohol yield was 1.23 g/L by using strain 10. The loss of substrate limonene is partly oxidized to (*R*)-(+)-perillyl alcohol, partly oxidized to other by-products such as perillyl aldehyde, and partly evaporated throughout the reaction process.

## Discussion

Whole-cell biocatalyst is commonly used to produce multiple compounds (Goldberg et al., 2007; Fukuda et al., 2008; Hou et al., 2015). The reasons for this are multifold. Whole-cell catalyst offers enhanced operability and economy, relative to extracted enzyme. Moreover, it provides better stability and can be repeatedly used in bioconversions, without loss of activity (Richter et al., 2010). Furthermore, whole-cell catalyst is more tolerant to substrate and provides higher product yield than extracted enzyme (Li et al., 2018).

During whole-cell biotransformation, low activity enzyme negatively affects (*R*)-(+)-perillyl alcohol production. Hence, it is crucial to balance enzyme levels during biosynthetic reactions. In this study, we employed varying plasmid copy numbers to regulate enzyme levels, as was previously reported (Wu et al., 2013; Hou et al., 2017). CymA overexpression in pRSFDuet-1 (high copy number) yielded high enzymatic activity and enhanced (*R*)-(+)-perillyl alcohol synthesis in strain 03 (Rico et al., 2019). (*R*)-(+)-perillyl alcohol synthesis by strain 01 (pACYCDet-2-*cymA*) and strain 02 (pET28a (+)-*cymA*) was slightly lower than strain 3 (Figure 2B and Supplementary Table S2). CymA carries the genes *cymAa* and *cymAb*, which encode the two subunits (hydroxylase subunit and reductase subunit) of p-cymene monooxygenase (Eaton, 1997). It is possible that the hydroxylase subunit cymAa was strongly expressed in strain 03 with the high copy plasmid.

We also demonstrated that different alkL expressions were constructed in strains 04, 05, and 06. Strain 04, producing the lowest alkL levels, maintained the stability of enzymatic activity for 2 h (Figure 2B). Strain 06, on the other hand, produced the highest alkL levels, which lead to whole-cell biocatalyst toxicity and reduced production (Figure 2B). In contrast, two-liquid phase biotransformation with whole cell catalyst (strain 04), featuring low alkL levels, enhanced biocatalyst viability and stability, thereby enhancing product titers. Julsing et al. (2012) similarly reported that well-regulated alkL production and low alkBGT expression facilitates a 4.3 times increase in product titers in a two-liquid phase bioprocess. These evidences suggest the critical role of a balance between biocatalyst stability and maximal titiers in determining hydroxylation efficiency of whole-cell-based reactions. Here, we achieved remarkable enhancement of biocatalyst stability *via* fine-tuning alkL expression, and thus, maximizing (*R*)-(+)-perillyl alcohol production.

One of the important factors of (*R*)-(+)-perillyl alcohol production *via* biotransformation is reaction condition. We established the optimal pH and temperature of whole-cell biocatalyst to be pH 7.4 and 20°C (Figures 4A,B). In addition, our (*R*)-(+)-perillyl alcohol production was 130 mg/L, when 50 OD_600_ whole-cell catalyst and 20 g/L substrate concentration was employed (Figures 4D,E). In prior studies, (*R*)-(+)-perillyl alcohol was shown to be crucial for whole-cell biotransformation at elevated substrate concentration. Limonene causes *E. coli* growth reduction during fermentation at concentrations between 1.05 and 3.36 g/L (Dunlop et al., 2011; Chubukov et al., 2015; Edgar et al., 2017). It is possible that the inhibitory concentration of limonene on cells was increased by using high concentration whole cell catalysis and, thus, the yield was effectively augmented. Owing to its remarkable tolerance to elevated substrate concentration, our developed biocatalyst became efficient in whole cell bioconversion.

The representative cofactor NADH plays a central role in the conversion of (*R*)-(+)-limonene to (*R*)-(+)-perillyl alcohol (Agullo et al., 2017). (*R*)-(+)-perillyl alcohol synthesis was augmented by 1.2 times with 2 mM NADH, as opposed to no NADH addition (Figure 4C). NADH regeneration, based on formate dehydrogenase, is highly developed (Alissandratos et al., 2014). Given that NADH is expensive ($260 per gram) (Britton et al., 2018), we expressed *fdh* from *Candida boidinii* to construct strain 10. Recently, 230 mg/L (*R*)-(+)-perillyl alcohol was produced using whole cells (OD_600_ = 50) of strain 10 during batch biotransformation. FDH is also used in the synthesis of multiple other compounds (L-gulose, aminobutyric acid) to achieve NADH regeneration (Chen et al., 2021; Zhang et al., 2021). At present, the formate dehydrogenase activity is not very strong, therefore, it is necessary to screen other dehydrogenases (GDH) or modify enzymes (Zhu et al., 2017).

Sufficient oxygen is required for the hydroxylation of monoxygenase, but not NADH (Li et al., 2002). This was supplied with aeration in the bioreactor. Finally, 20 g/L(*R*)-(+)-limonene was catalyzed to produce 1.23 g/L (*R*)-(+)-perillyl alcohol, with a substrate conversion rate 6% (Figure 5). In two-liquid phase bioconversion, 20–30% (*R*)-(+)-limonene was lost *via* evaporation, and the remaining 70–80% was oxidized (Figure 5). The loss of (*R*)-(+)-limonene was increased with the prolongation of conversion time. Hence, excess substrate was sacrificed. The production was not very obvious with the addition of 5 g/L (*R*)-(+)-limonene at 12 h (date not known). It is possible that (*R*)-(+)-perillyl alcohol was converted to (*R*)-(+)-perilla aldehyde (Figure 5). CymA converts p-cymene to a combination of p-cumic alcohol and p-cumic aldehyde (Eaton, 1997). So, it is likely that there were (*R*)-(+)-perillyl alcohol and (*R*)-(+)-perilla aldehyde mixtures in the fermentation products. In order to further enhance production, directed evolution of cymA or deletion of alcohol dehydrogenase [conversion of (*R*)-(+)-perillyl alcohol into (*R*)-(+)-perilla aldehyde] was carried out.

## Conclusion

Here, we demonstrated that recombinant *E. coli* expressing cymA can synthesize (*R*)-(+)-perillyl alcohol, with whole cell catalysis. High plasmid copy number expressing cymA is highly beneficial to (*R*)-(+)-perillyl alcohol production, and low alkL levels promote whole cell stability. We optimized catalytic conditions, as well as cell and substrate concentration, to further enhance (*R*)-(+)-perillyl alcohol production. Simultaneously, we also achieved NADH cofactor regeneration. Finally, (*R*)-(+)-perillyl alcohol production in strain 10 was 1.23 g/L in a 5 L bioreactor, and improved by 82 times, compared to strain 01 (15 mg/L). The whole cell catalysis approach could be useful for various valuable chemicals and pharmaceuticals.

## Data Availability

The original contributions presented in the study are included in the article/Supplementary Material, further inquiries can be directed to the corresponding authors.

## Abbreviations

CymAa, p-cymene monoxygenase hydroxylase; CymAb, p-cymene monoxygenase reductase; CymA, p-cymene monoxygenase hydroxylase and p-cymene monoxygenase reductase; FDH, formate dehydrogenase; alkL, outer membrane protein; GDH, glucose 1-dehydrogenase.
