# Supplementary material for: Efficient Synthesis of (R)-(+)-Perillyl Alcohol From (R)-(+)-Limonene Using Engineered Escherichia coli Whole Cell Biocatalyst
Source: Front Bioeng Biotechnol. 2022 Apr 25;10:900800. doi: 10.3389/fbioe.2022.900800 (PMC9084310; doi:10.3389/fbioe.2022.900800)
Supplement: Supplementary file 1 [file DataSheet1.PDF]

*Supplementary material*

**Efficient synthesis of (*R*)-(+)-perillyl alcohol from (*R*)-(+)-limonene using engineered *Escherichia coli* whole-cell biocatalyst**

Chao Sun[1,2], Rubing Zhang[2,*], Congxia Xie[1,*]

[1]A State Key Laboratory Base of Eco-Chemical Engineering, College of Chemistry and Molecular Engineering, Qingdao University of Science and Technology, Qingdao, China

[2]CAS Key Laboratory of Bio-based Materials,Qingdao Institute of Bioenergy and Bioprocess Technology, Chinese Academy of Sciences, Qingdao, China

*Corresponding author.

Rubing Zhang

CAS Key Laboratory of Bio-based Materials, Qingdao Institute of Bioenergy and Bioprocess Technology, Chinese Academy of Sciences, Qingdao, China

E-mail: zhangrb@qibebt.ac.cn

Congxia Xie

A State Key Laboratory Base of Eco-Chemical Engineering, College of Chemistry and Molecular Engineering, Qingdao University of Science and Technology, 53 Zhengzhou Rd., Qingdao 266042, China.

E-mail: xiecongxia@126.com

**Table S1. Primers employed for plasmids construction.**

| Primers | Sequences(5'-3') |
|---|---|
| pET28a(+)-*cymA*-F | TAGGTTCCGATTTAGTGCTTTACGGCACCTCGA |
| pET28a(+)-*cymA*-R | CAGTCACGTTAGCGATAGCGGAGTGTATAC |
| pRSFDuet-1-*cymA*-F | TCATCACCACAGCCAGGATCCGATGTGGGAGTACATCAAATATTACTTTG |
| pRSFDuet-1-*cymA*-R | ACCTGCAGGCGCGCCGAGCTCTTAGCTATTACGGCCATCGGTG |
| cymA-5 | TAAGACGTCCCCGGGTTATAATAATAC |
| pACYCDuet-2-*cymA*-F | CACCACAGCCAGGATCCGATGTGGGAGT |
| pACYCDuet-2-*cymA*-R | GACCTGCAGGCGCGCCGAGCTCTTAGCTATTACGGCC |
| pACYCDuet-2-lac-*alkL*-F | TAATAAGGAGATATACCATGAGCTTTAGCAACTATAAAGTGATTG |
| pACYCDuet-2-lac-*alkL*-R | ACCTGCAGGCGCGCCGAGCTCTTAAAACACATAGCTCGCGCC |
| pACYCDuet-2-trc-*alkL*-F | ATAAGGAGGAATAAACCATGAGCTTTAGCAACTATAAAGTGATTG |
| pACYCDuet-2-trc-*alkL*-R | TTCTTTACCAGACTCGAGCTCTTAAAACACATAGCTCGCGCC |
| pACYCDuet-2- *alkL*-F | TAATAAGGAGATATACCATGAGCTTTAGCAACTATAAAGTGATTG |
| pACYCDuet-2- *alkL*-R | ACCTGCAGGCGCGCCGAGCTCTTAAAACACATAGCTCGCGCC |
| pRSFDuet-1-*cymA*-*fdh*-F | CCGATGGCCGTAATAGCTAAAAAAGGAGATATACCATGAAGATCGTT |

| | |
|---|---|
| pRSFDuet-1-*cymA*-*fdh*-R | ACCTGCAGGCGCGCCGAGCTCTTATTTCTTATCGTGTTTACCGTAAGC |

**Table S2 Specific hydroxylation activity of whole cells of three strains different copy numbers**

| Strains | Copy numbers | Specific activity (U $g_{CDW}^{-1}$) |
|---|---|---|
| strain 01 | 10 | 2.56±0.16 |
| strain 02 | 40 | 2.89±0.22 |
| strain 03 | 100 | 3.81±0.24 |
